# Marine Invertebrate Xenobiotic-Activated Nuclear Receptors: Their Application as Sensor Elements in High-Throughput Bioassays for Marine Bioactive Compounds

**DOI:** 10.3390/md12115590

**Published:** 2014-11-24

**Authors:** Ingrid Richter, Andrew E. Fidler

**Affiliations:** 1 Environmental Technology Group, Cawthron Institute, Private Bag 2, Nelson 7012, New Zealand; E-Mail: andrew.fidler@cawthron.org.nz; 2 School of Biological Science, Victoria University of Wellington, P.O. Box 600, Wellington 6140, New Zealand; 3 Maurice Wilkins Centre for Molecular Biodiscovery, University of Auckland, Auckland 1142, New Zealand; 4 Institute of Marine Science, University of Auckland, Auckland 1142, New Zealand

**Keywords:** xenobiotic, nuclear receptor, bioassay, marine, bioactive, invertebrate

## Abstract

Developing high-throughput assays to screen marine extracts for bioactive compounds presents both conceptual and technical challenges. One major challenge is to develop assays that have well-grounded ecological and evolutionary rationales. In this review we propose that a specific group of ligand-activated transcription factors are particularly well-suited to act as sensors in such bioassays. More specifically, xenobiotic-activated nuclear receptors (XANRs) regulate transcription of genes involved in xenobiotic detoxification. XANR ligand-binding domains (LBDs) may adaptively evolve to bind those bioactive, and potentially toxic, compounds to which organisms are normally exposed to through their specific diets. A brief overview of the function and taxonomic distribution of both vertebrate and invertebrate XANRs is first provided. Proof-of-concept experiments are then described which confirm that a filter-feeding marine invertebrate XANR LBD is activated by marine bioactive compounds. We speculate that increasing access to marine invertebrate genome sequence data, in combination with the expression of functional recombinant marine invertebrate XANR LBDs, will facilitate the generation of high-throughput bioassays/biosensors of widely differing specificities, but all based on activation of XANR LBDs. Such assays may find application in screening marine extracts for bioactive compounds that could act as drug lead compounds.

## 1. Introduction

A major challenge facing researchers investigating marine natural products, with a view to identify potential drug lead compounds, is the selection and/or development of suitable bioassays. Typically the bioassays selected reflect the researcher’s long-term, applied science goals but often they have little ecological or evolutionary rationale. The goal of this review is to promote the idea that chemical detection mechanisms, which adaptively evolve to allow marine animals to detect dietary bioactive chemicals, can be used in bioassays for marine bioactive chemicals. More specifically, we propose that nuclear receptor (NR) proteins may provide “sensor elements” that can be utilized in bioassays. Briefly, in the envisaged bioassays the “sensor element” would be a NR ligand-binding domain (LBD), which binds a bioactive dietary chemical and the resulting conformational change is then transduced into an output signal.

Having stated the overall goal of this review, its limits should also be made explicit. First, the term “xenobiotic receptor” (XR) will be used in this review to denote members of the zinc-finger NR transcription factor super-family that are activated by xenobiotic chemicals (xenobiotic, from the Greek *xenos*: foreigner; *bios*: life). Under this definition we have excluded genuine xenobiotic receptors such as the aryl hydrocarbon receptor (AhR), which are activated by xenobiotics but do not belong to the NR super-family because they bind DNA by a different mechanism [1,2,3]. In principle, such non-NR xenobiotic receptors could also be utilized in similar high-throughput screens to those proposed here but this is beyond the scope of this review. To remove any ambiguities in terminology, the term “xenobiotic-activated nuclear receptor” (abbreviated XANR) will hereafter be used for the group of xenobiotic receptors considered in this review [4,5,6].

**Figure 1:**
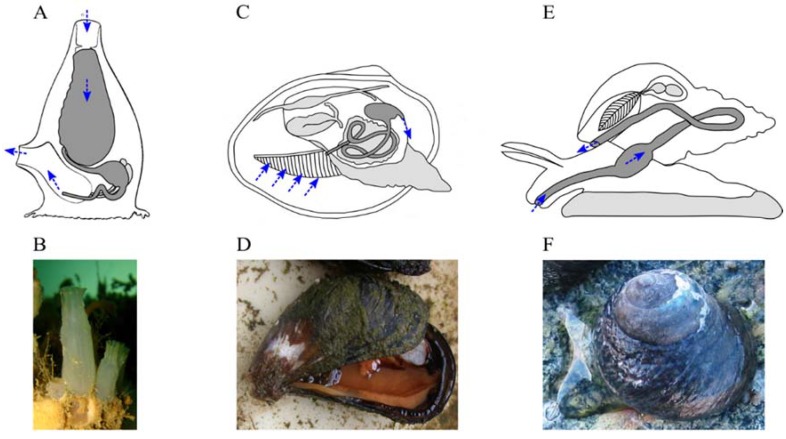
Examples of filter-feeding and surface-grazing marine invertebrates. Marine invertebrates that filter their diet from seawater are found in a range of taxonomic groups including the phyla *Chordata* and *Mollusca* (**A**–**D**). In contrast, grazing gastropods (Phylum *Mollusca*) scour their food from surfaces (**E**,**F**). Whatever their ecological niche, marine invertebrates require detoxification mechanisms in order to detect, and effectively metabolize, any potentially deleterious xenobiotics encountered in their diet (**A**,**C**,**E**). Schematic diagrams of the digestive tracts (dark grey) of filter-feeding tunicates (**A**), filter-feeding bivalve molluscs (**C**), and grazing gastropod molluscs (**E**) are shown and the direction of food movement is indicated by blue arrows. Examples of a filter-feeding tunicate (**B**), *Ciona intestinalis*, Phylum *Chordata*; a filter-feeding bivalve mollusc (**D**), *Mytilus edulis*, Phylum *Mollusca*; and a grazing gastropod mollusc (**F**), *Amphibola crenata*, Phylum *Mollusca*.

Marine invertebrate XANRs will be the primary focus of this review as such organisms occupy diverse ecological niches and are characterized by great taxonomic diversity. Significantly, this diversity includes both filter-feeders and intense surface-grazers, two foraging behaviors that expose animals to dietary xenobiotics at high concentrations (Figure 1). For example, it is well-established that filter-feeding marine invertebrates clear large volumes of phytoplankton from the water column and consequently bioaccumulate high concentrations of microalgal biotoxins [7,8]. It is to be expected that selective pressures associated with any bioactive compounds, particularly toxic ones, ingested by marine invertebrates may drive adaptive evolution of XANR LBDs that bind these ingested compounds. This idea is explored further in this review.

## 2. Bioactive Chemicals are Naturally Present in Animal Diets

Chemicals in animal diets are often viewed as simply energy sources (e.g., carbohydrates, lipids), building blocks (e.g., proteins), or biochemical pathway intermediates (e.g., vitamins). However, it is apparent that some dietary chemicals have “physiological” roles in the sense that they modulate animal biochemistry and physiology. The biological effects of such dietary chemicals range from influences on reproductive physiology and developmental transitions through to acute poisoning [9,10,11,12,13,14]. Animal taxa exposed to bioactive dietary xenobiotics evolve both behavioral and physiological traits to minimize any associated deleterious effects [15]. Many animals simply avoid eating plants/prey likely to contain toxins with such avoidance behaviors being both instinctual and learned [16,17]. For example, vertebrates tend to avoid bitter-tasting plants, as many poisonous phytochemicals (e.g., alkaloids) taste bitter [18,19,20]. Interestingly, there is evidence that natural selection associated with bitter taste perception may have influenced the evolution of bitter taste receptor gene repertoire sizes in vertebrate genomes [20,21,22]. In the marine environment, avoidance of toxic/unpalatable prey by coral reef fish is well-documented [23,24,25], while bivalve molluscs can limit their exposure to toxic compounds using behavioral responses, such as shell closure and restriction of filtration rate [8,26,27,28]. Despite avoidance behaviors, the diet of many animals will inevitably contain bioactive, and potentially toxic, chemicals that need to be metabolized and eliminated from their bodies [29,30,31,32,33].

## 3. Detoxification Pathways and Their Transcription-Level Regulation

Metazoan organisms have specialized biochemical pathways that metabolize and eliminate potentially toxic chemicals, whether endogenously synthesized or exogenously acquired. The complexities of detoxification biochemistry are beyond the scope of this review and are outlined in a number of recent reviews [34,35,36,37,38]. Nonetheless, a brief overview of metazoan detoxification pathways, and the transcriptional control of associated genes, is required to understand the functions of XANRs [39,40].

Detoxification pathways have been classified into three functional stages: oxidation/reduction (Phase I), conjugation (Phase II), and elimination (Phase III) [41,42]. It is to be expected that the genes encoding the functional elements of all three phases may be under both conservative and, at times, adaptive evolutionary pressures reflecting variation in the structures and mode of action of different xenobiotics/toxins associated with different animal diets [33,43]. The cat family (*Felidae*) provides an example of the apparent consequences of relaxation of conservative selective pressures associated with reduced ingestion of phytochemicals. Both domesticated and wild members of the *Felidae* are extremely susceptible to poisoning by phenolic compounds. This sensitivity is associated with a mutation in the feline orthologue of the gene encoding the enzyme UDP-glucuronosyltransferase (UGT) 1A6, a Phase II phenolic compound detoxification enzyme, leading to pseudogenization [44,45]. It is speculated that such UGT1A6 inactivation mutations are tolerated in the *Felidae*, and other hypercarnivores, because of relaxed selective pressures associated with their diet [46], as hypercarnivores are rarely exposed to plant-derived phenolic compounds [45].

In the context of this review, it is the *control of transcription* of detoxification related genes that is of particular interest. Expression of many detoxification pathway genes can be induced by the xenobiotic(s) that the pathway ultimately metabolizes [38,42,47]. Such xenobiotic-mediated induction of gene expression is of medical interest because of its implications for the efficacy, persistence, and side-effects of therapeutic drugs [34,48,49]. Xenobiotic-mediated induction of detoxification gene expression is best characterized for Phase I cytochrome P450 (CYP) enzymes, particularly members of CYP sub-families CYP1-4 that are associated with xenobiotic metabolism [34,48,50,51]. For example, levels of the human CYP enzyme CYP3A4, an enzyme responsible for oxidizing >50% of medicinal drugs, are induced by a range of therapeutic compounds, such as rifampicin, tamoxifen, and hyperforin [49,52], while human CYP1A2 enzymatic activity is induced by polycyclic aromatic hydrocarbons (PAHs) [34,53]. Interestingly, there exists striking inter-taxa variation in inductive responses to some xenobiotics [54]. For example, the steroidal drugs pregnenolone 16α-carbonitrile (PCN) and dexamethasone are highly efficacious CYP3A enzyme inducers in rodents but not so in humans [55,56]. In contrast, rifampicin is a strong inducer of human and dog CYP3A but not of rodent CYP3A [57,58]. While none of these examples are likely to be of ecological/evolutionary significance, such conspicuous inter-taxa variation in the response to xenobiotics suggests the possibility of adaptive evolution in the genetic elements that control expression of detoxification genes.

Xenobiotic induction of cytochrome P450 enzyme levels has also been reported in several invertebrate phyla, most prominently in the *Arthropoda* in the context of pesticide resistance [59,60,61]. In insects, the CYP enzymes induced by xenobiotics mainly belong to the sub-families CYP4, CYP6, and CYP9 [36]. In both *dipteran* and *lepidopteran* insect taxa, the barbiturate phenobarbital induces CYP enzymatic activity in association with transcription level induction of the CYP4, CYP6, and CYP9 genes [62,63,64,65]. In the honey bee (*Apis mellifera*), aflatoxin and propolis, ecologically relevant natural xenobiotics, induce CYP gene expression [66,67], while in the “model” arthropod *Drosophila melanogaster* many CYP genes are induced by caffeine and phenobarbital [68,69,70]. In *Drosophila mettleri* the CYP4D10 gene is induced by primary host plant alkaloids but not by similar alkaloids from a rarely utilized host plant. Clearly this finding suggests adaptive evolution of the associated gene induction mechanism(s) of *Drosophila mettleri* [71]. The soil nematode *Caenorhabditis elegans* (phylum *Nematoda*) displays up-regulated CYP gene expression in response to exposure to multiple xenobiotics [72,73,74]. For example, beta-naphthoflavone, polychlorinated biphenyl, PCB52, and lansoprazol are all strong inducers of almost all *C. elegans* CYP35 isoforms [72]. Rifampicin, one of the strongest inducers of the human CYP3A4 gene, is also a strong inducer of the *C. elegans* CYP13A7 gene [75]. Amongst marine invertebrates the polychaete *Perinereis nuntia* (phylum *Annelida*) shows increased levels of some CYP gene transcripts after exposure to benzo[α]pyrene (BaP) and PAHs [76]. In an ecologically more relevant context, the marine gastropod *Cyphoma gibbosum* (phylum *Mollusca*) is suggested to have adapted to feeding exclusively on highly toxic gorgonian corals by differential regulation of transcripts for two CYP enzymes, CYP4BK and CYP4BL [77].

Whilst most xenobiotic-mediated gene induction research has focused on Phase I CYP genes, Phase II glutathione S-transferases (GST) and Phase III multidrug resistance-associated proteins (MRPs) have also been reported to be inducible by some xenobiotics [35,78]. For example, expression of mouse GSTA1, MRP2, and MRP3 genes is induced by both pregnenolone 16α-carbonitrile (PCN) and 1,4-Bis[2-(3,5-dichloropyridyloxy)]benzene (TCPOBOP) [79]. In the marine environment, dietary toxins (e.g., cyclopentenone prostaglandins) have been shown to be both inducers and substrates of GST enzymes in three marine molluscs [80,81,82].

In summary, it is likely, that xenobiotic-mediated *control* of detoxification pathway gene expression may evolve adaptively in response to the differing chemicals to which different animals are exposed to, with diet probably being the main route of exposure [66,67,77,83]. The next section will consider the role of a specific group of ligand-activated transcription factors, the XANRs, in xenobiotic-mediated control of gene expression and speculate how natural selection may influence the adaptive evolution of such XANR genes.

## 4. Xenobiotic Receptors: Functions, Structures, and Taxonomic Distribution

### 4.1. Vertebrate Pregnane X Receptor

Functional characterization of XANRs is most advanced in a few selected vertebrate taxa [6,84,85,86]. Mammalian genomes encode two XANRs: constitutive androstane receptor (CAR; NR notation: NR1I3) and pregnane X receptor (PXR; NR notation: NR1I2). Although both CAR and PXR regulate the transcription of genes involved in detoxification of endogenous and exogenous (*i.e.*, xenobiotic) chemicals [85,86,87], PXR is the better understood with respect to how its LBD structure relates to ligand-binding and subsequent activation [85]. Therefore, the following sections of this review will focus on PXR and its orthologues.

Mammalian PXR was originally identified in genomic sequence data and designated as an orphan NR as its ligand(s) were then unknown. In 1998, three groups independently reported mammalian PXR activation by both steroids and a range of xenobiotics resulting in three alternative receptor names—with pregnane X receptor (PXR) now the most widely used [88,89,90]. PXR appears to function much like a standard ligand-activated NR. After ligand-binding within the PXR LBD, the activated PXR protein forms a complex with retinoid X receptor (RXR) before translocating from the cell cytoplasm into the nucleus. The PXR/RXR heterodimer binds to appropriate DNA response elements, thereby influencing transcription of adjacent genes [91,92,93,94]. Many of the PXR regulated genes are involved in detoxification. Thus, PXR activation, following xenobiotic-binding to its LBD, provides a mechanistic link between the presence of xenobiotics in a cell and appropriate detoxification gene expression [95].

**Figure 2:**
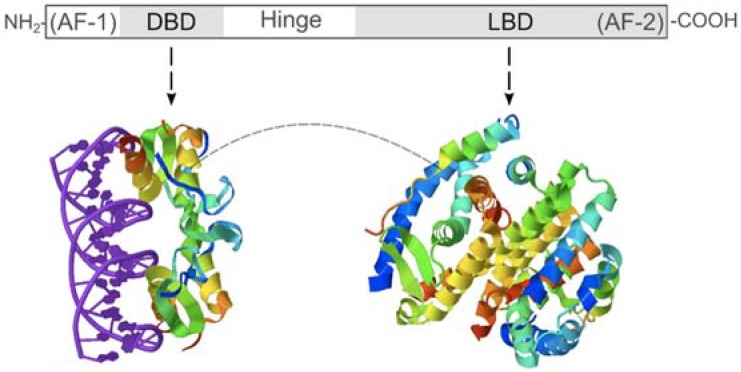
Schematic summary of the generic nuclear receptor (NR) structure. The overall structure of ligand-activated NRs is conserved through evolution with five key structural domains: *N*-terminal transcription activation domain (activation function-1, AF-1), DNA-binding domain (DBD), flexible hinge region (Hinge), and ligand-binding domain (LBD), which includes a *C*-terminal activation domain (activation function-2, AF-2). The DBD interacts with DNA while the structurally separate LBD interacts with ligands [106].

Vertebrate PXR ligands include a structurally diverse range of endogenously produced molecules (e.g., bile acids, steroid hormones, and vitamins) along with exogenously acquired chemicals (e.g., both synthetic and herbal drugs) [96,97,98,99,100,101]. Determination of the three dimensional structure of the human PXR protein has helped explain its striking permissiveness with respect to the differing structures of activating ligands. The classic model of NR function proposes that NR ligand specificity arises from the interaction between ligands and a ligand-binding domain (LBD) of the NR protein (Figure 2). In the majority of NRs the LBD cavities have well-defined shapes with restricted mobility, thereby ensuring specificity of ligand—NR LBD interactions [102,103]. In contrast, the human PXR LBD is both larger than is typical of NRs and also displays significant flexibility during ligand binding allowing it to accommodate a wider range of ligand sizes and structures [104,105].

Vertebrate PXRs display greater inter-taxa variation in LBD sequences than is typical of NRs, along with some evidence of positive selection within the LBD [4,107,108,109]. From this observation it has been speculated that such inter-taxa PXR LBD sequence differences may reflect adaptive evolutionary changes which enhance PXR binding of those exogenous dietary bioactives/toxins typically encountered by an organism within its particular ecological niche [4,5,107,108,110,111]. Notwithstanding such speculation, it has been shown experimentally that the differential activation of human and rat PXRs by some ligands can be attributed, in part, to specific residues within the PXR LBD [88,89,112,113,114,115,116]. For example, rifampicin is an effective activator of human PXR but has little activity on rat PXR [112] and this difference can be attributed to differences between the two PXR LBDs at a single position: human PXR Leu_308_/rat PXR Phe_305_ [117].

### 4.2. Non-Marine Invertebrate XANRs

Although most advances in our understanding of XANR function and evolution have been within the *Vertebrata*, there has been some progress in characterizing invertebrate XANRs, building on the conceptual foundations provided by the vertebrate PXR studies. It should be noted that phylogenetic approaches to identifying XANR genes within the genomes of non-chordate invertebrate taxa face significant challenges, the biggest being the large evolutionary distances separating the functionally characterized vertebrate query sequences typically used to search invertebrate genomes. The scale of this challenge is reinforced by considering that the split between the deuterostome (includes vertebrates) and protostome (includes most invertebrates) lineages occurred approximately one billion years ago [118,119,120]. Fortunately, model invertebrate organisms within two protostome phyla, *Arthropoda* (*D. melanogaster*) and *Nematoda* (*C. elegans*), provide the experimental route of using mutant animal phenotypes to assess the functions of putative XANRs initially identified on the basis of sequence homologies [121,122]. A PXR/NR1I-like homologue, termed hormone receptor-like 96 (*HR96*), identified in the *D. melanogaster* genome was found to be selectively expressed in tissues involved in the metabolism of xenobiotics [121,123]. *D. melanogaster* flies homozygous for Dhr96 null alleles displayed increased sensitivity to both phenobarbital and the pesticide 1,1,1-trichloro-2,2-di(4-chlorophenyl)ethane (DDT) along with defects in phenobarbital induction of gene expression [121]. These findings are consistent with experiments, using a combination of RNA interference treatments and *Cyp6d1* promoter reporter assays, that indicate a role for *DHR96* in mediating phenobarbital associated gene induction in *Drosophila* Schneider (S2) cells [123]. In summary, Dhr96 represents a strong candidate as a *bona fide* arthropod XANR and it may also have roles in cholesterol homeostasis and lipid metabolism [124,125]. A DHR96 orthologue from a freshwater aquatic arthropod (*Daphnia pulex*), DappuHR96, has been shown to be activated by a structurally diverse range of both endogenously produced compounds and xenobiotics, consistent with a role as a lipid and/or xenobiotic sensor [126]. Probable *DHR96* orthologues have also been identified in a range of other arthropod genomes *albeit* with no reports of their functional characterization: beetles [*Tribolium castaneum*] [127], ants [*Camponotus floridabus*] [128], honey bee [*Apis mellifera*] [129], and fall armyworm [*Spodoptera frugiperda*] [130].

The genome of the model nematode *C. elegans* (phylum *Nematoda*) encodes an exceptionally large number of NRs (~284) [131]. Responses of mutant *C. elegans* strains to toxin exposure indicates that one of these NRs, denoted NHR-8, a homologue of *D. melanogaster* HR96, is involved in the regulation of detoxification enzyme induction which is consistent with NHR-8 functioning as a XANR [122].

From a phylogenetic perspective one useful generalization emerges. To date, all arthropod and nematode putative, and partially functionally verified, XANRs are placed in the NR1J group of the NR super-family [126]. NR1J forms a sister group of the NR1I sub-family, which contains all the chordate XANRs. This grouping is suggestive of a shared ancestral XANR gene being present in the genome of a common ancestor preceding the divergence of the protostome-deuterostome lineage [132].

### 4.3. Marine Invertebrate XANRs

Over a decade ago, phylogenetic analyses identified two orthologues of vertebrate PXR encoded in the genome of a marine invertebrate chordate; the solitary tunicate *Ciona intestinalis* [133,134,135,136,137]. As the functional characterization of one of these *C. intestinalis* putative XANRs is central to this review, the associated experiments will be described in more detail in Section 5. More recently, two putative PXR/NR1I orthologues have been detected in the genomic sequence of the colonial tunicate *Botryllus schlosseri* [138], while the genome of the pelagic tunicate *Oikopleura dioica* encodes as many as six NR1I clade genes [139].

Although NR encoding genes can be confidently identified in a growing number of publicly available non-chordate marine invertebrate genomic sequences [140], recognizing *bona fide* XANRs within the NR repertoire remains problematic. To date, all functionally characterised XANRs, both deuterostome and protostome, have been placed in the NR1 group of the NR super-family (deuterostome: NR1I; protostome: NR1J) [89,114,121,123,126,141,142,143]. Therefore, at present, any predicted marine invertebrate NRs placed in the NR1 group may be regarded as potential XANRs. However, such phylogeny-based designations are always highly tentative and only functional data can lead to the confident assignment of a NR1 protein as a functional XANR (Section 5).

Despite the evident phylogenetic/bioinformatic challenges, candidate XANR genes have been identified in sequence data derived from a number of non-chordate marine invertebrates. For example, at least one of the two NRs identified in a demosponge (*Amphimedon queenslandica*, Phylum *Porifera*) genome displays functional characteristics consistent with a role in detecting xenobiotics [144,145]. The genome of the starlet sea anemone (*Nematostella vectensis*, Phylum *Cnidaria*) encodes three NR genes equally related to sub-families NR1 and NR4, while the genome of the Pacific oyster (*Crassostrea gigas*, Phylum *Bivalvia*) encodes as many as 23 NR genes placed in the NR1 sub-family [146,147]. In contrast, the genome of a marine deuterostome, the sea urchin (*Strongylocentrotus purpuratus*, Phylum *Echinodermata*), appears to lack NR1I sub-family genes although three NR1H genes were identified [148]. The apparent absence of NR1I subfamily genes from the S. *purpuratus* genome should be treated with caution as it may simply reflect an incomplete data-set [148].

We suggest that functional characterization of at least one molluscan XANR would be a very useful advance, as both filter-feeding (*Bivalvia*) and grazing (*Gastropoda*) marine molluscs are expected to be exposed to a wide range of bioactive chemicals through their diet (Figure 1) [7,149]. It is also worth noting that molluscs provide a cautionary tale warning against assumptions that sequence homology/orthology necessarily predict shared function. For example, the molluscan homologue of the vertebrate steroid receptors is a constitutive, rather than a ligand-activated, transcription activation factor [150].

In summary, although significant bioinformatic challenges remain, it is clear that the ever increasing nucleotide sequence databases provide an informational resource in which putative marine invertebrate XANRs can be identified with varying degrees of confidence. However, functional characterization of such putative XANRs is always needed, as will be described in the next section.

## 5. Marine Invertebrate Putative XANR LBDs are Activated by Known Marine Bioactive Compounds

In 2002, the solitary tunicate *Ciona intestinalis* was the first marine invertebrate to have an assembled and annotated genome sequence published [137,151]. Analysis of the *C. intestinalis* genomic sequence, in combination with *C. intestinalis* expressed sequence tag (EST) databases, revealed two genes that phylogenetic analyses placed as orthologous to vertebrate NR1I genes. These two *C. intestinalis* NR1I-like genes were denoted “VDR/PXR” reflecting their putative orthology with both the vertebrate PXR and vitamin D receptor (VDR) genes [137,152]. Hereafter, these two *C. intestinalis* genes will be abbreviated as *Ci*VDR/PXRα (GenBank accession number: **NM_001078379**) and *Ci*VDR/PXRβ (**NM_001044366**) [153,154]. At the time of writing there is no functional data published regarding *Ci*VDR/PXRβ so it will not be discussed further here.

Functional characterization of *Ci*VDR/PXRα began with its LBD being expressed, as part of a chimeric protein, in a mammalian cell line. Briefly, the *Ci*VDR/PXRα LBD was joined to the generic GAL4 DNA-binding domain (GAL4-DBD) and the resulting chimeric protein was shown to mediate ligand-dependent expression of a luciferase reporter gene in mammalian cells [153,155]. Using this mammalian cell line bioassay, an extensive range of both natural and synthetic chemicals (*n* = 166) were screened for their activity [153,155] and three putative *Ci*VDR/PXRα LBD agonists were identified (6-formylindolo-[3,2-b]carbazole: EC_50_ = 0.86 μM; *n*-butyl-*p*-aminobenzoate: EC_50_ = 16.5 μM; carbamazepine: EC_50_ > 10.0 μM). Based on these results a pharmacophore model was tentatively defined which consisted of a planar structure with at least one off-center hydrogen bond acceptor flanked by two hydrophobic regions [153,156]. Note that none of the three *Ci*VDR/PXRα LBD agonists identified were strikingly potent, having EC_50_ values in the μM range, nor did it seem plausible that these chemicals would have been encountered by *C. intestinalis* over evolutionary time.

**Figure 3:**
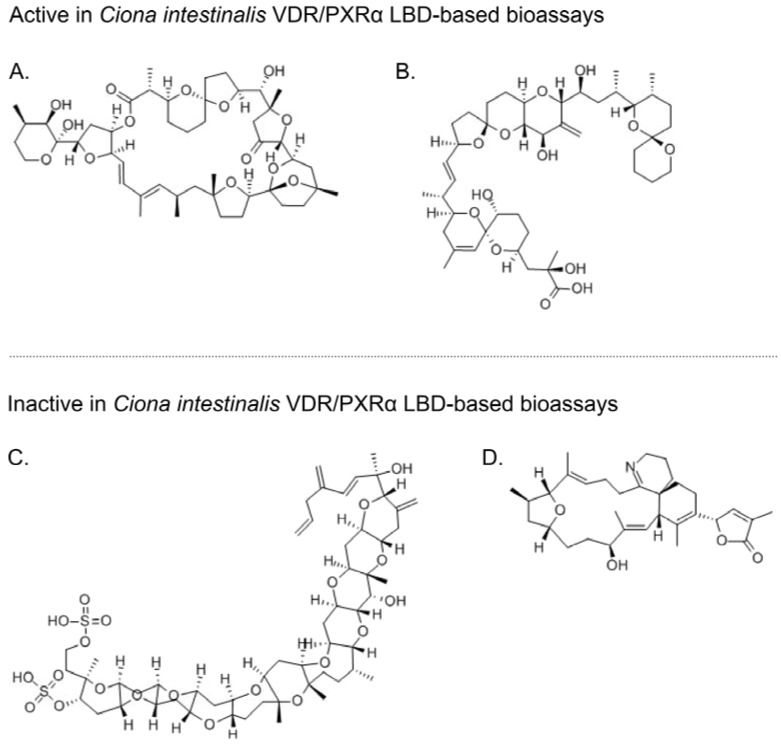
Structures of four microalgal biotoxins tested for activation of *C. intestinalis* VDR/PXRα LBD-based bioassays. Pectenotoxin-2 and okadaic acid activated bioassays that used the *C. intestinalis* VDR/PXRα LBD as the sensor element while yessotoxin and gymnodimine did not. (**A**) pectenotoxin-2 (CID: 6437385); (**B**) okadaic acid (CID: 446512); (**C**) yessotoxin (CID: 6440821); and (**D**) gymnodimine (CID: 11649137). *Abbreviations*: CID, PubChem compound accession identifier [157].

**Figure 4:**
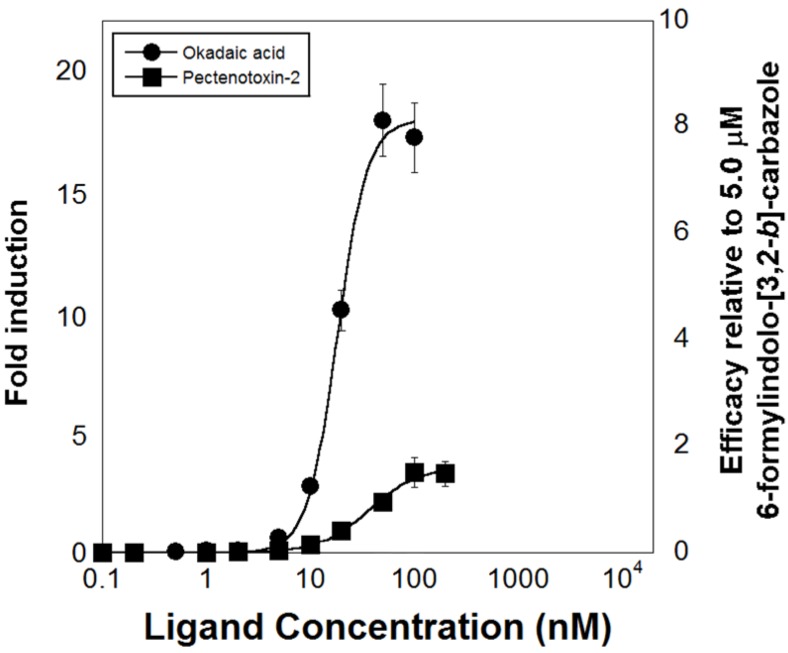
Microalgal biotoxin concentration-dependent response curves of luciferase expression induction by cell lines transfected with GAL4-DBD-*Ci*VDR/PXRαLBD fusion genes. The doubly-labelled ordinate axes indicates fold induction compared to vehicle control (left axis) and efficacy relative to 5.0 mM 6-formylindolo-[3,2-*b*] carbazole (adapted from Fidler *et al.* [156], with permission from © 2014 Elsevier).

Pursuing the hypothesis that the natural ligands of *Ci*VDR/PXRα include marine bioactive compounds frequently present in a marine filter-feeder’s diet, four microalgal biotoxins (okadaic acid, pectenotoxin-2 (PTX-2), gymnodimine, and yessotoxin) were tested for activation of the *Ci*VDR/PXRα LBD [156] (Figure 3). The four microalgal biotoxins investigated have diverse structures and all came with the caveat that their toxicity towards intact tunicate animals was, and still is, unknown. Of the four biotoxins tested, okadaic acid (EC_50_ = 18.2 nM) and PTX-2 (EC_50_ = 37.0 nM) activated the bioassay, while gymnodimine and yessotoxin were inactive [156] (Figure 4). Interestingly, the EC_50_ values for okadaic acid and PTX-2 are in the low-to-mid nM range making these ligands two to three orders of magnitude more potent than the three synthetic compounds previously found to be active in the *Ci*VDR/PXRα LBD-based bioassay [153].

In summary, the combined studies of Ekins *et al.* (2008) and Fidler *et al.* (2012) established that the *Ci*VDR/PXRα LBD displayed xenobiotic/ligand-binding characteristics consistent with *Ci*VDR/PXRα having a role in detecting bioactive marine chemicals naturally encountered by filter-feeding marine invertebrates through their diet [153,156]. Interestingly, the *Ci*VDR/PXRα LBD appears to have rather narrow ligand selectivity when compared to vertebrate, particularly human, PXRs [153,156]. It is possible that such tunicate VDR/PXR LBD ligand selectivity may reflect tunicate genomes encoding multiple VDR/PXR paralogues, with each paralogue subtype perhaps binding a differing range of ligand structures [138,139]. It should also be remembered that, despite the insights obtained from *Ci*VDR/PXRα LBD functioning in mammalian cell lines, the actual role of VDR/PXR orthologues in intact, living tunicates has not been determined and this represents an important area of future research. Nonetheless, the critical point is that the work of Ekins *et al.* (2008) and Fidler *et al.* (2012) firmly established the feasibility of using marine filter-feeder XANR LBDs as “sensors” in bioassays for marine bioactives. How this basic concept might be developed into cost-effective, high throughput bioassays will be considered next.

## 6. Development of High-Throughput Bioassays Based on Marine Invertebrate XANR LBDs

### 6.1. XANR LBD-Based Bioassays: Technical Considerations and Challenges

As outlined in Section 5, it is established that marine invertebrate XANR LBDs can be utilized as “sensors” in bioassays for marine bioactive chemicals. However, significant technical challenges exist for expanding this simple insight into economically and technically viable bioassays for high-throughput screening of marine compounds. Fortunately, existing NR LBD-based assays provide a strong foundation to build on. This section outlines how well-established NR LBD-based approaches could be applied to marine invertebrate XANR LBDs [158,159].

#### 6.1.1. NR LBD Bioassays Using Recombinant Yeast

Mammalian cell line-based bioassays, as described in Section 5, have the advantage that they provide a cellular/biochemical milieu shared by metazoan cells, which is expected to assist correct folding/functioning of proteins expressed from heterologous genes. However, mammalian cell lines do have significant limitations as they require costly, highly specialized culturing facilities and associated laboratory skills. In contrast, baker’s yeast (*Saccharomyces cerevisiae*) provides a well-established eukaryotic expression system that is inexpensive and suitable for standard microbiology laboratories. Furthermore, the yeast’s nutrient requirements are easily met in a 96-well plate format making *S. cerevisiae*-based bioassays well-suited to high-throughput screening formats [160,161,162]. In addition, *S. cerevisiae* cells do not contain endogenous NRs to potentially interfere with bioassays based on introduced NRs [163,164]. Finally, *S. cerevisiae* offers the possibility of directed evolution of NRs whereby NR LBD variant sequences, generated by *in vitro* mutagenesis, can be selected for enhancement of growth rates in the presence of a cognate ligand [165]. For example, Chen and Zhao (2003) combined random *in vitro* mutagenesis together with directed evolution to generate novel variants of the human estrogen receptor alpha (ERα) LBD that had significantly modified ligand-binding properties [165].

Despite the clear attractiveness of *S. cerevisiae* cells for NR LBD-based bioassays, there are aspects of NR functioning that require consideration when designing any associated high-throughput bioassays. Bioassay design needs to address how ligand-induced conformational changes in an introduced metazoan NR LBD will be transduced into a quantifiable output signal. Particular consideration needs to be given to the step in the NR signal transduction pathway selected for activation of the “output signal”. Two ligand-dependent steps in a NR signal transduction pathway have been utilized: (i) ligand-dependent binding of the NR to co-activator protein(s) [166] or (ii) ligand-dependent binding of the NR/co-activator complex to a specific promoter control region [167]. Indeed approach (i) has been successfully used for detecting ligand-dependent interactions between the human PXR and its co-activator protein, human steroid co-activator-1 (hSRC-1) [159,168]. Despite this success with human PXR, it is clear that this approach requires extensive knowledge of a given NR’s co-activator protein, knowledge, which generally will not be available for most marine invertebrate XANRs. Approach (ii) utilizes binding of ligand-activated NRs to control sequences in reporter gene promoters as the mechanism to generate an “output signal” [169,170]. For native, full-length marine invertebrate XANRs to be used in this approach, both the XANR’s co-activators (if any) and the sequences of the cognate DNA elements to which the XANR DBD binds need to be known. Again, for marine invertebrate XANRs such specialized knowledge is unlikely to be available. Even when putative response elements for metazoan XANRs have been identified in marine invertebrate genomes [171], these may not function as required in *S. cerevisiae* cells. Fortunately, such knowledge gaps can be bypassed by exploiting the highly modular structure of NRs (Figure 2). Basically a chimeric protein can be generated in which the XANR LBD is fused to the generic GAL4-DBD, which is native to yeast cells, removing any need for knowledge of the natural heterodimer partners of the XANR or the DNA sequence elements to which the XANR binds through its native DBD.

Following binding to well-characterized DNA control elements, via the GAL4-DBD, the XANR’s ligand-dependent activation domain (AF-2) (Figure 2) needs to function within the nuclear milieu of yeast cells. As previous studies have shown that the AF-2 domains of some vertebrate NRs do not function in yeast cells [172,173], a generic transcription activation domain (AD) from the *Herpes simplex* virion protein 16 (VP16) can be added to the C-terminus of the chimeric proteins [173]. In summary, a fusion gene can be generated encoding a chimeric protein that contains the GAL4-DBD, the XANR LBD, and the VP16-AD, with the ligand-binding characteristics of the chimeric protein determined by the XANR LBD.

The reporter gene selected to generate the “output” signal from yeast-based bioassays must combine low background with a clear response signal following activation of the NR LBD. In addition, it is highly desirable that the assay used to quantify reporter gene expression is non-lethal, allowing repeated measurements to be taken over time. Three types of NR-dependent reporter gene assays have been used in recombinant yeast: the *Escherichia coli lacZ* gene, encoding the enzyme β-galactosidase [174], yeast-enhanced green fluorescence protein (yEGFP) [163,175,176], and the luciferase gene [177]. Although the luciferase and yEGFP reporter assays have been shown to be somewhat more sensitive than *lacZ* [178] both have associated complications. For example, luciferase assays require the use of expensive substrates and involve cell lysis [174,179] which can be problematic either due to released cellular proteases [174] or incomplete cell lysis [179]. Although yEGFP assays do not require the addition of substrates, the assays are characterized by a high natural background of green fluorescence [163]. In contrast, *lacZ* assays are inexpensive and, when based on the chromogenic substrate (chlorophenol red-β-d-galactopyranoside, CPRG), do not require cell lysis [180]. Such non-lethal measurement of β-galactosidase activity is useful as it means that repeated measurements can be taken over time. This is a significant advantage because the time course of *lacZ* gene transcription induction will vary between ligands due to differences in parameters such as membrane permeability and solubility in the cytoplasm [160].

Notwithstanding the technical challenges many metazoan NRs have been successfully expressed in *S. cerevisiae* in combination with reporter genes [162,166,168,170,181,182,183]. Furthermore, some of the resulting yeast strains have found application in screening environmental samples for bioactivities—particularly for estrogenic activity [176,180,183,184,185,186,187]. Thus, it is well-established that *S. cerevisiae* provides a suitable expression system for bioassays in which a NR LBD acts as the sensor element that interacts with bioactive chemicals to be detected [183,185,187].

Despite the clear merits of recombinant yeast-based NR bioassays it remains true that all cell-based bioassays face limitations intrinsic to living cells. Such limitations include test compounds being unable to cross cell membranes or being directly toxic to the cells themselves. To address such limitations biosensor techniques have been developed to directly measure physical interactions between macromolecules and their potential ligands.

#### 6.1.2. Biosensors for High-Throughput Screening for NR LBD Ligands

During the past two decades biosensors, which measure a range of physiochemical changes associated with interactions between molecules, have been developed within both industry and academia [188,189]. The main advantage of such biosensors is their cell-free nature thereby removing some of the limitations associated with cell-based bioassays, such as the need for test compounds to cross cell membranes [160]. Biosensors typically consist of a macromolecule immobilized on a surface via either covalent or strong non-covalent bonds [190]. An important consideration is that such attachments should not significantly influence the natural structure of the macromolecule or change its functioning in unpredictable ways [190]. Following immobilization, a wide range of techniques exist to detect and quantify interactions between the immobilized macromolecules and potential ligands—including calorimetric, acoustic, electrical, magnetic, and optical sensing techniques [189].

Numerous NR LBD-based biosensors have been developed, principally in the context of drug development [166,190]. Among the established xenobiotic receptors, the human PXR LBD has been successfully used as the sensor element in a number of differing biosensor formats [111,191,192]. Such biosensors have confirmed previously known human PXR ligands such as hyperforin, clotrimazole, ginkgolide A, SR12813, and 5b-pregnane-3,20-dione [111,112,191]. The successful development of human PXR LBD-based biosensors supports the theoretical feasibility of using marine invertebrate XANR LBDs in biosensor formats to screen for marine bioactive compounds. Furthermore, if routine production of correctly folded and soluble marine invertebrate XANR LBDs can be mastered, then they could be used in affinity chromatography to identify and isolate novel XANR ligands, as has recently been reported for the human PXR LBD [193].

### 6.2. XANR LBD-Based Assays: Biological, Ecological, and Evolutionary Considerations

The application of marine invertebrate XANR LBDs in high-throughput bioassays/biosensors entails some biological, ecological, and evolutionary considerations. The first point to emphasize is the vast taxonomic diversity of marine invertebrates, along with the myriad of ecological niches they occupy. It is expected that this diversity and complexity will be paralleled by the diversity and complexity of the bioactive xenobiotics to which these animals are exposed to during their life-cycles. If dietary bioactive xenobiotics do act as selective agents in shaping the structure of marine invertebrate XANR LBDs, then there exists an effectively unlimited supply of “sensors” in the sea that have been pre-molded by natural selection to facilitate detection of marine bioactive compounds.

If this perspective is correct, then a major decision confronting developers of marine invertebrate XANRs-based bioassays is how to select, from the virtually unlimited options, the marine invertebrate XANRs to use. We suggest that three considerations should both guide and restrict this decision. The first is the implicit assumption that a bioactive chemical that has acted as a significant selective pressure driving molecular evolution of a given marine invertebrate’s XANR LBD may also be active on human cells/tissues. In this context it could be argued that the more closely related an organism is to humans, the more similar would be their susceptibility to marine bioactive chemicals. Whilst doubtless a simplification, this idea does suggest that selecting XANR LBDs from marine invertebrate taxa within the phylum *Chordata* (tunicates, *Urochordata*; lancets, *Cephalochordata*; acorn worms, *Hemichordata*) would be a useful strategy. However, it should be emphasized that there is no *a priori* reason why taxon selection should be restricted to the *Chordata*. For example, at least one marine microalgal biotoxin has acted as a selective pressure in the evolution of bivalves (phylum *Mollusca*) while also being toxic to vertebrates [194,195]. In addition, selections may be restricted simply because promising marine bioactives may exist in contexts/ecological niches to which no marine invertebrate chordate is adapted. This highlights a second consideration in selecting marine invertebrate XANRs for bioassays. The ecological niche occupied by the taxa from which XANR LBDs could be isolated requires careful consideration as such niches restrict the xenobiotics influencing XANR LBD function and evolution. For example, filter-feeding bivalves use a somewhat different mechanism for filtering seawater than do filter-feeding tunicates—and therefore these two groups of filter-feeders ingest somewhat differing profiles/size-ranges of marine microorganisms [196]. It is also important to consider that some bioactive chemicals may be produced by marine organisms that adhere to hard surfaces. For example, benthic microalgae, such as the dinoflagellate *Gambierdiscus toxicus* can produce highly toxic compounds (e.g., ciguatera-associated toxins) [197]. To detect such toxins, XANR LBDs from surface-grazing animals would probably be more suitable for the bioassay than XANR LBDs from filter-feeding animals. A third consideration guiding XANR LBD selection is simply the genomic resources available. Clearly, when a specific marine invertebrate taxon has been selected on evolutionary and ecological grounds, then the required genomic datasets can be generated for increasingly realistic costs. Nonetheless, as discussed earlier in this review, bioinformatic challenges exist when designating a NR as a putative XANR based solely on homology/phylogeny. As a generalization, more reliable predictions of *bona fide* XANRs are likely within those phyla, particularly *Chordata* and *Arthropoda*, for which a number of functionally characterized XANRs exist.

## 7. Future Prospects for Marine Invertebrate XANR LBD-Based Bioassays

The discovery of useful natural marine bioactive compounds, along with the subsequent development of derived pharmaceuticals, faces immense technical challenges [198]. Consequently, despite the enormous number of structurally unique bioactive marine natural products that are now known, to date the associated pharmaceutical pipeline comprises only eight approved drugs, along with twelve natural marine products (or derivatives thereof) in different phases of clinical testing [198,199]. Obviously the natural biological activities of potential drug lead compounds influence their potential medical applications, so bioassay design is a major limiting factor in the detection of useful bioactives [198,200]. In this context we see potential for XANR LBD-based bioassays as their specificities rest on natural evolutionary processes that may have molded the XANR LBD’s structure and its associated ligand-binding properties. Thus, in a sense, these evolutionary processes can provide “creative input” into the bioassay design. Due to the highly modular structure of NRs the XANR LBDs can be combined with generic DNA-binding and transcription-activating domains in various cell-based bioassays or can be used as purified proteins in biosensors. By selecting, on the basis of taxonomy and ecology, the organism to source the XANR LBD from it may be possible to tailor bioassays to search for bioactive compounds from differing sources. As an example, LBDs of potential XANRs identified in the genomes of marine filter-feeding organisms like tunicates and bivalves could be used to test for bioactive compounds associated with the myriad of microorganisms that make up the diet of marine filter-feeders (Figure 1) [7,149].

## 8. Conclusions

This review began with the assertion that, from both an ecological and evolutionary perspective, many bioassays currently used to screen for marine bioactive chemicals are somewhat “arbitrary”, in the sense that they bring together chemicals and biological detection systems that would rarely, if ever, be combined in nature. To address this deficiency, we have proposed that members of a specific group of ligand-activated transcription factors—marine invertebrate xenobiotic-activated nuclear receptors (XANRs)—provide a source of bioassay sensor elements that have been pre-molded by natural selection for detecting bioactive chemicals present in marine invertebrate diets. As a proof-of-concept we outlined recent work showing that mammalian cell lines expressing tunicate XANR LBDs, coupled with an appropriate reporter gene, can detect established microalgal biotoxins. Based on such success with mammalian cell lines we suggest that recombinant yeast strains expressing XANR LBDs may provide low-cost, high-throughput bioassays. Alternatively, it may be possible to entirely remove the need for live cells and adapt biosensor technologies to look for marine chemicals that directly bind to XANR LBDs. Whatever the assay format and technology used, marine invertebrate genomes, each with its own ecological niche and evolutionary history, represent an increasingly accessible informational resource of XANRs that can be harnessed to identify the chemical treasures that are undoubtedly hidden in the sea [198,201].

## Abbreviations

AD: activation domain
AhR: aryl hydrocarbon receptor
BaP: benzo [*α*] pyrene
*Bs*VDR/PXRα: *Botryllus schlosseri* VDR/PXR orthologue α
CAR: constitutive androstane receptor
*Ci*VDR/PXRα: *Ciona intestinalis* VDR/PXR orthologue α
*Ci*VDR/PXRβ: *Ciona intestinalis* VDR/PXR orthologue β
CPRG: chlorophenol red-β-d-galactopyranoside
CYP: cytochrome P450 gene
DBD: DNA-binding domain
DDT: 1,1,1-trichloro-2,2-di(4-chlorophenyl)ethane
ER: estrogen receptor
GAL4: yeast DNA-binding transcription factor
GST: glutathione S-transferases
HR96: nuclear hormone receptor 96
LBD: ligand-binding domain
MRP: multidrug resistance-associated protein
NR: nuclear receptor
NR1I: nuclear receptor sub-family 1, class I
NR1J: nuclear receptor sub-family 1, class J
NR1H: nuclear receptor sub-family 1, class H
OA: okadaic acid
PAHs: polycyclic aromatic hydrocarbons
PTX-2: pectenotoxin-2
PCN: pregnenolone 16α-carbonitrile
PXR: pregnane X receptor
RXR: retinoid X receptor
SRC-1: steroid co-activator 1
TCPOBOP: 1,4-Bis[2-(3,5-dichloropyridyloxy)]benzene
VDR: vitamin D receptor
VP16: viral protein 16
XANR: xenobiotic-activated nuclear receptor
XR: xenobiotic receptor
